# The Roles and Interactions of *Porphyromonas gingivalis* and *Fusobacterium nucleatum* in Oral and Gastrointestinal Carcinogenesis: A Narrative Review

**DOI:** 10.3390/pathogens13010093

**Published:** 2024-01-20

**Authors:** Bing Wang, Juan Deng, Valentina Donati, Nabeel Merali, Adam E. Frampton, Elisa Giovannetti, Dongmei Deng

**Affiliations:** 1 Department of Medical Oncology, Amsterdam University Medical Center, Cancer Center Amsterdam, Vrije Universiteit, 1081 HV Amsterdam, The Netherlands; b.wang1@amsterdamumc.nl (B.W.); j.deng@amsterdamumc.nl (J.D.); valentina.donati@gmail.com (V.D.); e.giovannetti@amsterdamumc.nl (E.G.); 2 Unit of Pathological Anatomy 2, Azienda Ospedaliero-Universitaria Pisana, 56100 Pisa, Italy; 3 Minimal Access Therapy Training Unit (MATTU), Royal Surrey County Hospital, NHS Foundation Trust, Egerton Road, Guildford GU2 7XX, UK; n.merali@surrey.ac.uk (N.M.); adam.frampton@surrey.ac.uk (A.E.F.); 4 Department of Hepato-Pancreato-Biliary (HPB) Surgery, Royal Surrey County Hospital, NHS Foundation Trust, Egerton Road, Guildford GU2 7XX, UK; 5 Section of Oncology, Department of Clinical and Experimental Medicine, Faculty of Health Medical Science, University of Surrey, Guilford GU2 7WG, UK; 6 Fondazione Pisana per la Scienza, 56100 Pisa, Italy; 7 Department of Prevention Dentistry, Academic Center for Dentistry Amsterdam (ACTA), University of Amsterdam and Vrije Universitreit Amsterdam, 1081 LA Amsterdam, The Netherlands

**Keywords:** periodontitis, oral squamous cell carcinomas, colorectal cancers, pancreatic ductal carcinomas, bateria–host interactions, microbial interaction

## Abstract

Epidemiological studies have spotlighted the intricate relationship between individual oral bacteria and tumor occurrence. *Porphyromonas gingivalis* and *Fusobacteria nucleatum*, which are known periodontal pathogens, have emerged as extensively studied participants with potential pathogenic abilities in carcinogenesis. However, the complex dynamics arising from interactions between these two pathogens were less addressed. This narrative review aims to summarize the current knowledge on the prevalence and mechanism implications of *P. gingivalis* and *F. nucleatum* in the carcinogenesis of oral squamous cell carcinoma (OSCC), colorectal cancer (CRC), and pancreatic ductal adenocarcinoma (PDAC). In particular, it explores the clinical and experimental evidence on the interplay between *P. gingivalis* and *F. nucleatum* in affecting oral and gastrointestinal carcinogenesis. *P. gingivalis* and *F. nucleatum*, which are recognized as keystone or bridging bacteria, were identified in multiple clinical studies simultaneously. The prevalence of both bacteria species correlated with cancer development progression, emphasizing the potential impact of the collaboration. Regrettably, there was insufficient experimental evidence to demonstrate the synergistic function. We further propose a hypothesis to elucidate the underlying mechanisms, offering a promising avenue for future research in this dynamic and evolving field.

## 1. Introduction

The human microbiome consists of a diverse range of bacteria that play a vital role in maintaining the equilibrium between health and diseases [1,2]. Among them, the oral microbiome stands as the second most diverse and intricate ecosystem [3] residing in the oral cavity, which is the primary site of entrance into both the digestive and respiratory systems. The enlarged Human Oral Microbiome Database reports the presence of over 700 bacterial species involved in dynamic and intricate microbial interactions [4]. The oral microbiome has garnered increasing attention in recent years due to its potential implication for various health/disease conditions, not only inside the oral cavity but also at distant body sites [5,6].

The significant advancements in next-generation sequencing technology and bioinformatic tools have facilitated the exploration of the harmonious equilibrium among the microbiome, the host, and the environment. It has been revealed that the microbial community as a whole, rather than a few single microbes, maintains this equilibrium. In the healthy state, the microbiome and host establish a symbiotic relationship; whereas in the disease state, a dysbiosis environment promotes the prevalence of pathogenic species in a microbiome, ultimately contributing to the development of illnesses [7]. It was shown that the dysbiotic oral microbiome not only leads to oral infectious diseases, such as caries and periodontitis, but also plays an important role in the development of multiple systemic diseases, including cardiovascular disease, rheumatoid arthritis, Alzheimer’s disease, pulmonary disease, and cancer [8,9,10,11,12].

Cancer, which is characterized by uncontrolled cell growth and potential metastasis, remains a leading cause of mortality globally [13]. Traditionally, the etiological factors of cancer have been attributed to intrinsic factors, like genetic, environmental, and lifestyle components [14]. Recent discoveries in cancer research revealed that tumor growth might be affected by the dynamic interactions between all constituents inside the tumor microenvironment (TME) as well [15,16]. Within the TME, there are not only intricate signaling contacts between cellular and non-cellular factors but also reciprocal interactions involving microbial components [16,17]. It was believed that the microbiome could be operated as a powerful regulator inside the TME, thereby influencing the host (immune) responses [7,18].

Oral squamous cell carcinoma (OSCC) and gastrointestinal cancers, including colorectal cancer (CRC) and pancreatic ductal adenocarcinoma (PDAC), are among the malignancies that have been linked to the oral microbiome in an intriguing way [19]. It was hypothesized that oral microbes reach distant body sites via the circulatory system after processes such as mastication and routine oral hygiene practices, like teeth brushing and flossing [20]. The concept of the “oral-gut axis” was proposed to illustrate this connection [21]. For example, *Fusobacterium nucleatum*, which is a well-known Gram-negative pathogen for periodontal infections, has been implicated in the development of systemic disorders, including premature birth, inflammatory bowel disease, and CRC. Another periodontal pathogen, namely, *Porphyromonas gingivalis*, was found to be associated with all three types of cancers: OSCC, CRC, and PDAC [22,23]. Until now, ample studies have explored the role of oral microbes in the onset and development of cancers [6,19,24]. However, it is noteworthy that most studies focused on the role of a single bacterial species. It has been acknowledged that tumor tissues do not harbor a single bacterial species, but a multi-species microbial community that accommodates active bacterial interactions [25,26]. These bacterial interactions could change the formation of tumors. For example, Pustelny et al. demonstrated in a murine tumor model where mice were coinfected with the cystic fibrosis pathogen *Pseudomonas aeruginosa* and a strictly anaerobic bacterium *Veillonella parvula* had worse survival rates due to higher *P. aeruginosa* loads in tumor tissues compared with those coinfected with either bacterial species alone [27]. Lertpirlyapong et al. showed that gastric colonization with multi-species microbiota and the carcinogenic pathogen *Helicobacter pylori* in male mice leads to more invasive gastrointestinal intraepithelial neoplasia than colonization with *H. pylori* alone [28]. Although both studies were conducted in mice, the experimental evidence hints at the potential importance of microbial interaction in cancer development. Thus, the existing knowledge on the contribution of a single bacterial species to cancer progression is insufficient without considering the complex dynamics arising from interactions within the microbial community.

The oral pathogen *F. nucleatum* is known as a bridging bacterium that is able to coaggregate with various bacterial species, such as *P. gingivalis*, *Treponema denticola*, and *Prevotella intermedia* [29,30]. Similarly, the fimbriae of *P. gingivalis* can mediate the coaggregation with *Streptococcus gordonii*, *Veillonella* sp., and *Aggregatibacter actinomycetemcomitans* [31,32]. It was shown that *F. nucleatum* can enhance the invasion of human gingival epithelial cells by *P. gingivalis*, which might increase the transmission of *P. gingivalis* to other body sites in periodontitis patients [33]. In line with the potential role of microbial interaction in cancer development, we raise the following questions: Does the interaction between *F. nucleatum* and *P. gingivalis* have a synergistic influence on the development of cancer? Do these two bacterial species compete for resources and space inside a tumor site?

The aim of this review is to summarize the current knowledge on the association of oral pathogens *P. gingivalis* and *F. nucleatum* with local tumor OSCC and distant tumor CRC and PDAC and the underlying mechanisms. In particular, we aimed to explore clinical and experimental evidence on the interactions between these two pathogens that influence oral and gastrointestinal carcinogenesis. To this end, a literature search was conducted to classify previous studies that discovered the relationship between oral bacteria and cancer, notably, OSCC, CRC and PDAC. A comprehensive search strategy was developed, including the following terms: “oral microbiome”, “oral bacteria”, “*Porphyromonas gingivalis*”, “*Fusobacteria nucleatum*”, “cancer”, “oral squamous cell carcinoma”, “colorectal cancer”, “pancreatic ductal adenocarcinoma”, and “interaction”. The search was conducted in several databases, such as PubMed, ScienceDirect, and Google Scholar. Various levels of evidence were collected, including studies with in vitro and animal experiments, as well as clinical observational studies.

In the following sections, three distinct types of cancers, namely, OSCC, CRC, and PDAC, are initially introduced. This is followed by a summary of the association of oral pathogens *P. gingivalis* and *F. nucleatum* with these cancer types and the underlying mechanisms. Finally, clinical evidence and experimental evidence are provided to explore the interplay between *P. gingivalis* and *F. nucleatum* in these three cancer types and hypotheses regarding the underlying mechanisms are proposed.

## 2. OSCC, CRC, and PDAC

In this narrative review, we focused on the association between *P. gingivalis* and *F. nucleatum* with OSCC, CRC, and PDAC because these two bacterial species were frequently identified in these tumor sites. OSCC represents a local tumor environment where *P. gingivalis* and *F. nucleatum* normally reside, whereas CRC and PDAC represent the distant tumor sites where oral microbes might reach via circulation.

CRC is a malignancy that affects the colon and rectum, both of which are integral components of the gastrointestinal tract. CRC has significant heterogeneity, manifesting in diverse clinical outcomes, therapeutic responses, and morphological characteristics. Reprogrammed metabolism is a hallmark of CRC, and CRC cells are geared toward rapid proliferation, requiring nutrients and the removal of cellular waste in nutrient-poor environments [34]. CRC is a prevalent disease that ranks among the most frequently occurring cancers globally, along with an elevated mortality rate. Based on the GLOBOCAN—Global Cancer Statistics 2020 study, it was the third most often diagnosed cancer worldwide, accounting for 10% of cases. Additionally, it was shown to be the second leading cause of cancer mortality, responsible for 9.4% of deaths. It was projected that there will be an increase in the number of new CRC cases by 2040, reaching roughly 3.2 million cases. This anticipated rise in cases is expected to significantly affect the global healthcare system [35,36].

PDAC is the most common malignancy of the pancreas. The most often seen symptoms in individuals with PDAC include weight loss, abdominal discomfort, and jaundice [37,38,39]. It is an aggressive and harmful ailment, with only 26% of patients living one year after being diagnosed and the disease continues to exhibit a discouraging average 5-year survival rate of 12% [40,41]. Based on the GLOBOCAN—Global Cancer Statistics 2020 study, the global incidence of PDAC in the year 2020 reached a total of 495,773 newly diagnosed cases, while the number of fatalities attributed to this disease amounted to 466,003 [42]. As of 2023, PDAC is projected to become the second leading cause of cancer-related mortality worldwide, surpassing CRC and breast cancer [43]. The management of PDAC remains notably challenging, necessitating a concerted effort to advance our understanding of biomarkers and explore interdisciplinary strategies.

## 3. The Prevalence of *P. gingivalis* in OSCC, CRC, and PDAC

*P. gingivalis* is a well-known periodontal pathogen. It is a Gram-negative anaerobic bacterium associated with the onset and progression of periodontitis [44]. Previous studies showed that it can colonize malignant tissues in an oral cavity, such as OSCC, ESCC, and gingival carcinoma [45]. Sayehmiri et al. conducted a meta-analysis that revealed the presence of *P. gingivalis* is associated with a risk increase of more than 1.36-fold in the development of OSCC [46]. An excessive amount of *P. gingivalis* was identified as a potential risk factor for OSCC [47,48,49]. 

In addition to oral cancers, *P. gingivalis* was frequently related to cancers at other body sites, including esophageal cancer, lung cancer, CRC, and PDAC [50,51,52,53]. Ample clinical studies found high abundances of *P. gingivalis* in both tumor tissue and fecal samples of CRC patients, which were correlated with the onset of CRC and poor prognosis in patients. For example, in a cross-sectional study, Kerdreux et al. examined 247 CRC patients and 89 controls (stages I–IV). They found a significant increase in *P. gingivalis* levels in fecal samples of CRC patients compared with the healthy controls. *P. gingivalis* could be identified in the fecal samples of 2.6–5.3% of CRC patients [22]. In another cohort study, *P. gingivalis* was detectable in 10 out of 31 CRC tissue samples using quantitative polymerase chain reaction (qPCR). A higher prevalence of *P. gingivalis* was found in individuals with the latter phases of colonic carcinogenesis [35]. 

Similarly, a high prevalence of *P. gingivalis* in PDAC patients has been reported. A recent prospective cohort study of 361 individuals diagnosed with PDAC found that the presence of *P. gingivalis* was associated with a 59% rise in PDAC development. Results also show an imbalance in oral microbial composition occurred before the onset of the cancer [54]. Another prospective cohort study examined 405 individuals diagnosed with pancreatic cancer, together with 410 control subjects, and found that the individuals with high levels of antibodies against *P. gingivalis* had a greater than twofold increased risk of developing PDAC [55].

Overall, there is clear clinical evidence that a high level of *P. gingivalis*, either in an oral cavity, fecal samples, or tumor tissues, is associated with the development of all three types of cancers.

## 4. Mechanisms of *P. gingivalis* in Cancer Development and Chemoresistance

As a known periodontal pathogen, *P. gingivalis* employs many strategies to compromise tissue integrity and impair the host immune response. These strategies include the prevention of cell apoptosis, stimulation of cell proliferation, initiation of chronic inflammation, and generation of oncometabolites [56].

**Figure 1:**
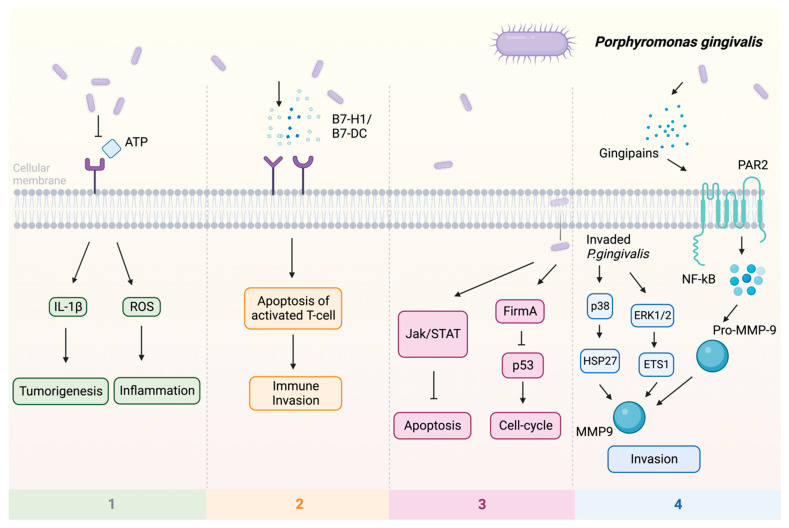
Multiple pathways employed by *P. gingivalis* in tumor induction: (1) P2X7 activation via ATP is blocked, leading to the stimulation of IL-1β, which promotes tumorigenesis, and the induction of ROS, which fosters a pro-inflammatory microenvironment. (2) Facilitation of immune evasion occurs through the activation of B7-H1 and B7-DC receptors, contributing to a (partial) circumvention of the immune system. Immune evasion is facilitated through the activation of B7-H1 and B7-DC receptors, contributing to a (partial) evasion of the immune system. (3) Activation of FimA results in the downregulation of p53, enhancing the host cell’s cell cycle while simultaneously suppressing apoptosis. The JAK/STAT axis is also implicated in the downregulation of apoptosis. (4) Additionally, *P. gingivalis* stimulates invasion through PAR2 activation via gingipains, activating NF-κB signaling, which leads to the formation of MMP-9, thereby enhancing *P. gingivalis* invasion. Upon invasion, pro-MMP-9 undergoes upregulation facilitated by ERK1/2 and ETS1, along with activation of p38 and HSP27. Abbreviations: ATP—adenosine triphosphate; ERK1/2—extracellular signal-regulated kinase 1/2; ETS1—protein; FimA—protein; HSP27—heat shock protein 27; IL-1β—interleukin-1β; JAK—Janus kinase 1; MMP-9—matrix metalloproteinase-9; NF-κB—nuclear factor kappa B; P2X7—purinergic receptor; pro-MMP-9—pro-matrix metalloproteinase-9; p38, p53—protein; ROS—reaction oxygen species; STAT—signal transducer and activator of transcription.

Previous studies indicate that *P. gingivalis* can promote tumorigenesis by influencing various signaling pathways (Figure 1): (1) Upon infection of the host by *P. gingivalis*, the B7-H1 receptor can be activated, facilitating the apoptosis of activated T cells. The increased expression of B7-H1 receptors in host cells may impact the persistence of inflammatory illnesses [57]. (2) Nucleoside Diphosphate Kinase (NDK), which is the effector protein produced by intracellular *P. gingivalis*, can block the signaling of extracellular adenosine triphosphate (ATP)/purinergic receptor (P2X7) on macrophages by consuming ATP. This prevents inflammasome activation and the secretion of interleukin-1β (IL-1β), consequently facilitating the process of tumorigenesis [58]. The NDK enzyme is also known to phosphorylate heat shock protein 27 (HSP27), and it is capable of triggering antiapoptotic processes upon phosphorylation [59]. (3) *P. gingivalis* activates antiapoptotic pathways, such as Janus kinase 1 (JAK1)/signal transducer and activator of transcription 3 (STAT3) and phosphoinositide 3-kinase PI3K/protein kinase B (Akt) in oral epithelial cells, thus promoting OSCC [60]. Besides inhibiting the intrinsic apoptosis of the invaded epithelial cells, *P. gingivalis* can also enhance the progression of the S phase of the cell cycle by inhibiting the p53 tumor suppressor gene (TSG) through the FimA adhesin [61]. Expression of the aforementioned B7-H1 receptor can inhibit the effector T cells by inducing regulatory T cells, which is beneficial for the invaded cell survival [57,62]. Due to the induction of regulatory T cells by the B7-H1 receptor, the immune system is (partly) evaded. (4) The activation of extracellular signal-regulated kinase 1/2 (ERK1/2)-protein ETS1, p38/HSP27, and PAR2 (protease-activated receptor 2)/nuclear factor kappa B (NF-κB) pathways was observed in response to *P gingivalis* infection, leading to the induction of pro-matrix metalloproteinase-9 (pro-MMP-9) expression, hence the increasing levels of MMP-9 and enhanced cellular invasion [63,64,65].

## 5. The Prevalence of *F. nucleatum* in OSCC, CRC, and PDAC

As mentioned above, *F. nucleatum* is another Gram-negative anaerobic bacterium often found in the resident oral microbiome and periodontal disease sites. It is known for its adhesive properties, which facilitate its attachment to other bacterial species and host cells [66]. Beyond its oral habitat, *F. nucleatum* was found in various cancer types, including CRC and PDAC [67,68,69]. Furthermore, its presence has been linked to worse survival rates in patients diagnosed with CRC and PDAC [70,71].

Extensive clinical investigations have reported a high prevalence of *F. nucleatum* in OSCC. A clinical cross-sectional study examined 80 paired OSCC tumors and adjacent normal tissues; *F. nucleatum* was detected in a striking 75.7% of OSCC tissue samples, contrasting with approximately 33.6% prevalence in normal samples, which is a reduction by 2.25-fold [68]. This significant difference highlights the potential diagnostic value of *F. nucleatum* in OSCC. The abundance of *F. nucleatum* in oral rinse samples has also been associated with the progress of OSCC. Yang et al. scrutinized the microbiota composition of oral rinses collected from a cohort of 51 healthy individuals and 197 patients diagnosed with OSCC at varying stages [72]. They found a notable increase in *F. nucleatum* abundance as oral cancer progressed. Its abundance increased from 2.98% in healthy controls to 4.35% in OSCC stage 1 and 7.92% in stage 4. These compelling findings underscore the potential relevance of *F. nucleatum* in both the onset and progression of OSCC.

Notably, *F. nucleatum* is a key member of CRC-associated bacteria. Multiple narrative review and systematic review articles summarized clinical evidence on the enrichment of *F. nucleatum* in CRC patients [70,73]. Generally, the abundance of *F. nucleatum* in CRC tumor tissues was found to be higher than in neighboring normal tissues [74]. Its abundance was also positively associated with CRC progression [75,76]. However, differential prevalence rates of *F. nucleatum* in CRC tissues have been reported. In particular, there was a notable disparity in the prevalence of *F. nucleatum* inside tumor tissues compared with normal tissues across different geographical cohorts, including the United States, Japan, and Europe. The occurrence of *F. nucleatum* in CRC tissues could vary from 13% to 75% [76]. Nevertheless, *F. nucleatum* has been considered a diagnostic and prognostic determinant in CRC patients [75].

The correlation between *F. nucleatum* prevalence and PDAC was not conclusive. Although Mitsuhashi et al. reported a detection rate of 8.8% for *Fusobacterium* species in 302 PDAC tissue specimens, with 283 positive and 25 negative detections of *Fusobacterium* species, Yamamura et al. did not detect any *F. nucleatum* in pancreatic cancer tissue [77,78]. Given these disparate findings, further in-depth analysis is warranted to elucidate the relationships between *F. nucleatum* and PDAC.

Similar to *P. gingivalis*, the high level of *F. nucleatum* is associated with the onset and progression of OSCC and CRC. But more clinical evidence is needed to clarify the association between *F. nucleatum* prevalence and PDAC.

## 6. Mechanisms of *F. nucleatum* in Cancer Development

Two major virulence factors of *F. nucleatum* are believed to be the most important factors in cancer progression [79,80]. The first factor is *Fusobacterium* adhesin A (FadA), which is also an important kinase in OSCC, induces oncogenic gene expression and promotes the growth of CRC cells; the other factor, namely, Fap2, which is derived from *F. nucleatum*, potentiates the progression of CRC by its inhibiting potential for immune cell activity through interacting with T-cell immunoreceptors with Ig and ITIM domains (TIGIT) [81,82]. Fap2 is unique to CRC but inactive in OSCC [83].

**Figure 2:**
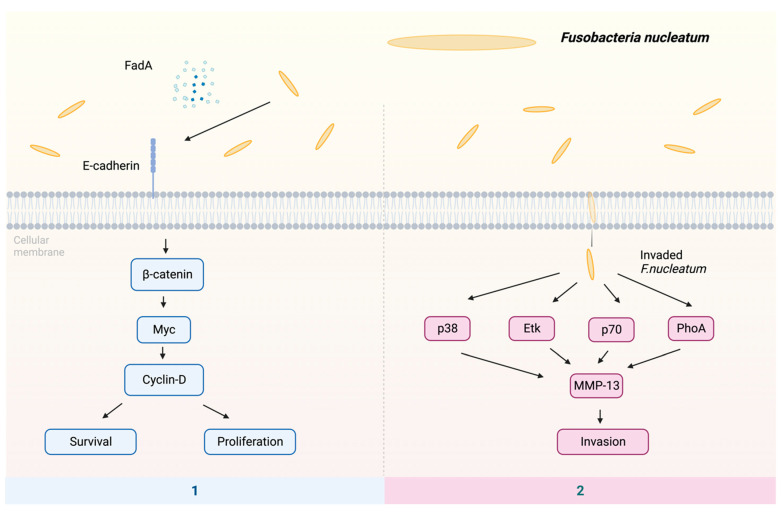
The potential mechanisms of *F. nucleatum* in cancer: (1) The virulence factor FadA binds to E-cadherin, subsequently activating β-catenin. This activation, in turn, triggers the transcription factor Myc, leading to the activation of cyclin-D. The activation of cyclin-D stimulates host cell survival and proliferation. (2) *F. nucleatum* enhances invasion by activating p38, Etk, p70, and RhoA, resulting in the upregulation of MMP-13. Abbreviations: Etk—tyrosine kinase; FadA—*Fusobacterium* adhesin A; MMP-13—matrix metalloproteinase-13; p38—protein kinase; p70—S6 kinase; RhoA—kinase.

The putative processes by which *F. nucleatum* may contribute to the development of malignancies include the following pathways (Figure 2): (1) The proliferation of oral epithelial cells is induced by *F. nucleatum* FadA [84,85]. Factor FadA binds to E-cadherin, which activates β-catenin [86]. The translocation of activated β-catenin from the cytoplasm to the cell nucleus leads to the production of oncogenes, such as Myc and Cyclin D, hence inducing cellular proliferation and upregulating the expression of oncogenic and inflammatory genes [83,87,88]. (2) The *F. nucleatum* infection can lead to an elevation in the synthesis of MMP-13, which is also known as collagenase 3, facilitating the movement of cells by activating Etk/BMX, S6 kinase p70, and RhoA kinase. In addition, *F. nucleatum* is able to activate mitogen-activated protein kinase p38, subsequently triggering the activation of heat shock protein-27 (HSP-27), leading to the production of MMP-9 and MMP-13 [89,90]. Remarkably, these enzymes are imperative in promoting tumor invasion and metastasis.

## 7. Coexistence of *P. gingivalis* and *F. nucleatum* in OSCC, CRC, and PDAC

As abovementioned, the association between *P. gingivalis*/*F. nucleatum* and cancer, including OSCC, CRC, and PDAC, and the underlying mechanisms have been extensively reviewed [19,81,84,86,91,92]. Many of these reviews summarized the role of *F. nucleatum* in the development of OSCC and CRC [81,84,86,91], whereas Saikia et al. and Irfan et al. discussed the involvement of both *P. gingivalis* and *F. nucleatum* in carcinogenesis [19,92]. We noticed that most reviews discussed the role of individual bacterial species in carcinogenesis. Although the interaction between *P. gingivalis* and *F. nucleatum* was mentioned in several reviews [19,84,86], the exact role and potential influence of this interaction on carcinogenesis were not extensively discussed. This current review attempted to summarize the clinical evidence on the co-existence of *P. gingivalis* and *F. nucleatum* in three cancer types. Furthermore, we highlight the intricate web of microorganisms that may influence the landscape of cancer. Table 1 summarizes the clinical studies that have reported the co-existence of *P. gingivalis* and *F. nucleatum* in patients with OSCC, CRC, and PDAC.

In oral infection diseases, such as periodontitis, the two pathogens *P. gingivalis* and *F. nucleatum* were often detected simultaneously [93,94]. Similarly, in OSCC patients, these two bacterial species were often detected simultaneously. In a cross-sectional study, Zhang et al. found that OSCC tissues were enriched with the *Fusobacterium* and *Porphyromonas* genera [95]. Torralba et al. reported significantly elevated levels of both *P. gingivalis* and *F. nucleatum* in OSCC tissue samples compared with those obtained from healthy subjects [96]. Furthermore, microbiota composition analysis demonstrated a marked increase in *P. gingivalis* and *F. nucleatum* abundance in tumor sites when compared with control groups. Chang et al. examined 61 cancer tissues, pericancerous tissues, subgingival plaque samples, and 30 normal tissues using qPCR. They revealed that both *P. gingivalis* and *F.* nucleatum existed at higher levels in cancer tissues than in normal tissues [45]. In addition, the relative number of these two bacterial species in cancer tissues positively correlated with that in subgingival plaque, indicating a link between periodontitis and OSCC. Compared with *F. nucleatum*, it seems that *P. gingivalis* infection was more positively associated with late clinical staging, low differentiation, and lymph node metastasis in OSCC patients. This phenomenon was observed in a cohort study by Park et al., where a higher serum level of *P. gingivalis* IgG was associated with a worse prognosis, even though the IgGs of both *P. gingivalis* and *F. nucleatum* were detected in OSCC patients [97].

The coexistence of *P. gingivalis* and *F. nucleatum* has also been reported for CRC patients. Several clinical studies found significant enrichment of both *P. gingivalis* and *F. nucleatum* in the tumor tissue, saliva, or fecal samples compared with healthy controls [49,98,99]. In an observational study by Purcell et al., the abundance of *P. gingivalis* and *F. nucleatum* were correlated with the consensus molecular subtypes (CMS) of CRC [49]. It was found that both bacterial species were strongly associated with CMS subtype 1 (CMS1), which is characterized by its inflammatory signatures, high mutation rate, and hypermethylation of CpG [100]. CMS1 tumors had a favorable prognosis when detected before metastasis but a poor prognosis after relapse. The enrichment of *P. gingivalis* and *F. nucleatum* in CMS1 might imply a potential microbial synergy within the tumor microenvironment. The results presented by Guven et al. confirmed the presence of *P. gingivalis* and *F. nucleatum* in saliva samples from CRC patients [98]. Gao et al. also found that the relative abundance of *P. gingivalis* and *F. nucleatum* was associated with CRC. Further microbial network analysis revealed that both genera *Porphyromonas* and *Fusobacterium* played central roles in inter-microbial interactions by being associated with other bacterial species in the microbial network [49]. *P. gingivalis* has been considered a keystone pathogen because it was able to alter oral microbiome composition and function, despite a low abundance [101,102]. It is likely that *P. gingivalis* and *F. nucleatum* act as key modulators for CRC tumorigenesis.

In PDAC patients, the evidence of the coexistence of *P. gingivalis* and *F. nucleatum* was inconclusive. In a case–control study, Fan et al. examined the microbial compositions of oral rinse samples collected from 361 PDAC patients and 371 healthy controls and found that the prevalence of *P. gingivalis* was associated with a higher risk of pancreatic cancer, but that of phylum *Fusobacteria* was associated with a decreased pancreatic cancer risk [54]. This observation was in line with the finding of a prospective cohort study where blood samples were taken from 405 pancreatic cancer patients and 416 matched controls. *P. gingvialis* was found to be twofold higher in the samples of cancer patients than those in the control. But there was no difference in the level of *F. nucleatum* between the cancer and control samples [55]. Conversely, Kartal et al. found elevated levels of *F. nucleatum* in the fecal samples of PDAC patients when compared with those of the controls [103]. However, *P. gingivalis* was not detected.

Generally, the clinical evidence showed that *P. gingivalis* and *F. nucleatum* co-existed in both OSCC and CRC. Higher levels of both bacterial species were associated with a worse prognosis. When going through the clinical evidence, we found that other bacterial genera or species than *P. gingivalis* and *F. nucleatum* were identified in tumor tissues or patients with tumors. This information is summarized in Section 8 below in order to give a glimpse of the complexity of the cancer-associated microbiome.

**Table 1:** Coexistence of *P. gingivalis* and *F. nucleatum* in OSCC, CRC, and PDAC samples.

Type of Cancer	Sample Types	Bacterial Detection Method	Pg, Fn ^a^	Major Bacterial Genera ^b^	References
OSCC	Tumor tissue	16S rRNA	Pg ↑, Fn ↑	*Streptococcus*, *Prevotella*, *Fusobacterium*	[95]
OSCC	Tumor tissue	16S rRNA	Pg ↑, Fn ↑	*Fusobacterium*, *Prevotella*, *Actinomyces*	[96]
OSCC	Tumor tissue	16S rRNA	Pg ↑, Fn ↑	*Capnocytophaga*, *Fusobacterium*, *Haemophilus*	[45]
OSCC	Serum	ELISA	Pg ↑, Fn ↑	*Porphyromonas*, *Fusobacterium*	[97]
CRC	Tumor tissue	16s rRNA	Pg ↑, Fn ↑	*Porphyromonas*, *Parvimonas*, *Peptostreptococcus*	[49]
CRC	Saliva	Real-time PCR	Pg -, Fn -	*Fusobacterium*, *Porphyromonas*, *Streptococcus*	[98]
CRC	Stool	Real-time PCR	Pg ↑, Fn ↑	*Porphyromonas*, *Fusobacterium*, *Bacteroides*	[99]
PDAC	Oral wash	16S rRNA	Pg ↑, Fn ↓	*Porphyromonas*, *Aggregatibacter*	[54]
PDAC	Fecal	16S rRNA	Pg: n. m, Fn ↑	*Faecalibacterium*, *Romboutsia*, *Bacteroides*	[103]

^a^: bacteria coexisting in specific type of samples; ^b^: top two to three bacterial genera with increased abundance within tumor tissues or patients; ↑: increased abundance in tumor tissues or patients; ↓: decreased abundance in tumor tissues or patients; -: no significant change; 16S rRNA: 16S ribosomal RNA gene amplicon sequencing; ELISA: enzyme-linked immunosorbent assay; real-time PCR: real-time polymerase chain reaction; Fn: *F. nucleatum*; Pg: *P. gingivalis*.

## 8. Complex Microbiome Related to OSCC, CRC, and PDAC

Table 1 also includes information on the top 2–3 major bacterial genera identified in clinical studies. Although it is not the goal of this review to provide a comprehensive overview of the compositions of the cancer-associated microbiome, the information in Table 1 highlights key aspects of the microbial interactions, emphasizing the presence of a diverse, multi-species community in the three tumor types. The findings from the listed clinical studies showed that multiple bacterial species, other than *P. gingivalis* and *F. nucleatum*, can be associated with cancer progression. There is no consensus on the tumor-specific bacterial genera. Up to 14 different bacterial genera are reported, which are enriched in either tumor tissue or cancer patients (Table 1). This evidence underlies the complex nature of the cancer-associated microbiome.

## 9. Interaction between *P. gingivalis* and *F. nucleatum* in Cancer Development

Although multiple clinical studies revealed the co-existence of *P. gingivalis* and *F. nucleatum* in cancer patients, the functions of this co-existence have not been well studied. We searched for various types of evidence in order to answer the following key questions: Do these two pathogens impose a synergistic effect on cancer development? Are there growth or niche competitions between these two bacterial species within a tumor site?

Using in vitro cell culture or murine models, researchers showed that the dual species of *P. gingivalis* and *F. nucleatum* can promote OSCC tumorigenesis via Toll-like receptors on the oral epithelial cells, leading to increased pro-inflammatory cytokine production, the epithelial–mesenchymal transition (EMT), and cell apoptosis inhibition [104,105,106,107,108]. Unfortunately, most studies did not include a *P. gingivalis*- or *F. nucleatum*-alone group as a control. It was impossible to identify whether there was a synergy between the two pathogens. So far, only two studies have compared the function of dual species with the corresponding single species alone [105,106]. Sztukowska et al. found that *P. gingivalis* alone induced the expression of transcription factor ZEB1 and promoted the migration of epithelial cells TGK1 in vitro [105]. Combining *F. nucleatum* or *Streptococcus gordonii* with *P. gingivalis* did not improve or inhibit the ZEB1 induction of *P. gingivalis*. Therefore, no synergy or competition between *P. gingivalis* and *F. nucleatum* was found in this experimental setting. Lee et al. confirmed that *P. gingivalis* alone can increase the expression of key EMT-promoting transcription factors, including Zeb1. But they found that the combination of *P. gingivalis* and *F. nucleatum* slightly enhances cell migration compared with each bacterial species alone [106]. Overall, there was insufficient experimental evidence to demonstrate any synergy between *P. gingivalis* and *F. nucleatum* in contributing to carcinogenesis. However, the coinfection of *P. gingivalis* and *F. nucleatum* has been reported in the field of periodontology [109]. Polak et al., using a rat model, revealed that the simultaneous presence of *P. gingivalis* and *F. nucleatum* resulted in a synergistic effect, leading to increased bone loss and intensified inflammatory reactions in the periodontal tissues compared with each pathogen alone [109]. Maekawa observed that the survival of intracellular *P. gingivalis* facilitated the intracellular survival of *F. nucleatum* in a coinfection subcutaneous chamber model [110]. Inversely, *F. nucleatum* can enhance the invasion of *P. gingivalis* into gingival epithelial cells [93]. This evidence from the field of periodontology indicates a possible collaboration between these two pathogens within a microbial community. Given the polymicrobial nature of the cancer-associated microbiome, it is critical to understand the microbe-induced tumorigenesis using dual- or multi-species coinfection models in the future.

## 10. Potential Mechanisms of *P. gingivalis*–*F. nucleatum* Co-Infection in Cancer Development

As mentioned above, there has been a lack of mechanistic studies that investigated the co-infection of *P. gingivalis* and *F. nucleatum* in in vitro or animal models. Hence, the potential roles of *P. gingivalis* or *F. nucleatum* in carcinogenesis have mainly been summarized based on single-species studies. Here, based on the existing knowledge, we propose our hypothesis on the mechanisms of the coinfections in cancer development.

It was suggested that the divergence in nutrient utilization could lead to the coexistence of *P. gingivalis* and *F. nucleatum*. For instance, *P. gingivalis* primarily degrades dipeptides, while *Prevotella* and *F. nucleatum* prefer smaller amino acids. This metabolic synergy is further underscored by *P. gingivalis*’ proteolytic nature, enabling it to provide amino acids to *F. nucleatum*, which lacks proteolytic capabilities. This mutualistic interaction fosters the colonization of additional *P. gingivalis*, thus creating a positive feedback loop, where more amino acids are supplied to other cohabiting bacteria [32]. The roles of single *P. gingivalis* and *F. nucleatum* in cancer development summarized in Figure 1 and Figure 2 indicate that these two bacterial species can utilize different pathways to function. *P. gingivalis* causes cancers via immune evasion, apoptosis inhibition, EMT, and establishing a chronic inflammatory state, while *F. nucleatum* promotes the occurrence and development of cancer through localization, proliferation, immune suppression, and metastasis [56,89]. Hence, it is possible that the co-infection of *P. gingivalis* and *F. nucleatum* can enhance tumorigenesis compared with single-species infection. The diverse mechanisms employed by both *P. gingivalis* and *F. nucleatum* pose challenges to the development of targeting therapeutics since multiple targets should be taken into account.

## 11. Potential Mechanisms of Microbiome in Cancer Development

Increasing evidence has pointed out that cancer development does not depend on the abundance of individual or several bacterial species, but is modulated by the function of an entire microbial community, which consists of hundreds of bacterial species. How a microbiome causes the onset and progression of cancer has not been systematically studied. Several hypotheses were proposed, including the driver–passenger model and cancer–microbiome–immune axis theory [111,112]. Although the effect of the cancer-associated microbiome was not the focus of this review, we highlight two hypotheses in order to increase awareness of the microbiome in mechanistic research.

The “driver-passenger” hypothesis was initiated in 2012 by Tjalsma et al. while aiming to explain the striking differences in CRC-associated microbiome compositions reported by various studies [111]. It proposes that pathogenic bacteria like *Bacteriodes* spp. can initiate CRC development and function as a driver. The driver-induced changes in the tumor microenvironment and cellular metabolism provide a competitive advantage to the passenger bacteria, which are opportunistic pathogens, such as *Fusobacterium* or *Streptococcus* spp. Eventually, the passengers can replace the drivers and subsequently either suppress or promote CRC progression. This hypothesis acknowledges the dynamic changes of microbiomes and suggests that the microbiome “snapshots” obtained from numerous clinical cross-sectional studies may not fully capture the comprehensive dynamics of the microbiome throughout the development of CRC.

According to Tjalsma, the driver–passenger hypothesis is unique to CRC and cannot be generalized as a microbial etiology [111]. Recently, Al-Hebshi et al. adapted the “driver-passenger” hypothesis and proposed a “passenger-tuning-driver” model to illustrate the role of microbiomes in OSCC [113]. Different from the “driver-passenger” model, it was believed that the oral microbiome was not involved in OSCC initiation. The tumor microenvironment selected or enriched the initial “passenger”. As it matures, the intra-tumor microbiome can turn into a functional “driver” by expressing pro-inflammatory components and virulence factors, consequently, enhance the OSCC progression.

The two models above emphasize the dynamic interaction between the microbiome and the host. The cancer–microbiome–immune axis concept proposed by Jain et al. includes the aspect of immunity, which explains the interplay between the microbiome, immunity, and cancer [112]. The microbiome can affect the tumor cells directly by serving as antigens, or indirectly by adjuvant signals, which lead to immunomodulation. The adjuvant signals can be sent in the form of various microbial-secreted products, such as metabolites, toxins, and vesicles, or cytokines secreted through the manipulation of host cells. The understanding of the cancer–microbiome–immune axis brings up new ideas for cancer therapy through microbial modulation. Modulation of the microbiome can be harnessed to potentiate the efficacy of immunotherapies and decrease their toxicity. So far, antibiotics, probiotics, and prebiotics have been developed for microbiome modulation [114,115,116]. But the actual clinical efficacy is yet to be improved.

It is important to acknowledge certain limitations of this narrative review. The clinical studies cited for demonstrating the co-existence of *P. gingivalis* and *F. nucleatum* in cancer patients were mostly cohort studies, which are relatively low in the hierarchy of evidence. To confirm the association between the co-existence of these two bacterial species and cancer development, a systematic review based on randomized control trials is necessary. Furthermore, this review focused only on the interaction between *P. gingivalis* and *F. nucleatum*. As shown in Table 1, other bacterial genera might be involved in the onset and progression of cancer. Within the complex microbial community, various microbial interactions might modulate the structure and function of the community, and hence, influence the process of carcinogenesis. Future research on the collective impact of bacterial consortia on cancer-related processes is poised to reveal novel insights into the complex relationship between the oral microbiome and tumors developed in both the oral cavity and at distant/internal body sites.

## 12. Conclusions

Within the aforementioned limitations, this review demonstrates the association of *P. gingivalis* and *F. nucleatum*, alone or together, in the initiation and development of OSCC, CRC, and PDAC. Based on the results of clinical studies, the prevalence of both bacteria species correlated with cancer development progression, emphasizing the potential impact of the collaboration. Regrettably, there was insufficient experimental evidence to demonstrate the synergistic function. Since the existing reported underlying mechanisms were based on single-species *P. gingivalis* or *F. nucleatum* studies, we propose that the *P. gingivalis*–*F. nucleatum* interaction might provide colonization advantages for both bacterial species. The diverse pathways employed by both bacterial species might enhance their pathogenicity and complicate the therapeutic targets. A deeper understanding of the microbial interplay may hold the potential to unlock innovative strategies for cancer management, underscoring the feasibility of targeted modulation of the microbiome to alter cancer trajectories.

## Data Availability

Not applicable.
